# Restraint Stress Inhibits Mouse Implantation: Temporal Window and the Involvement of HB-EGF, Estrogen and Progesterone

**DOI:** 10.1371/journal.pone.0080472

**Published:** 2013-11-14

**Authors:** Li-Hua Zhao, Xiang-Zhong Cui, Hong-Jie Yuan, Bo Liang, Liang-Liang Zheng, Yu-Xiang Liu, Ming-Jiu Luo, Jing-He Tan

**Affiliations:** College of Animal Science and Veterinary Medicine, Shandong Agricultural University, Tai-an City, P. R. China; South China Agricultural University, China

## Abstract

It is known that psychological stress affects reproduction in women, but it is unknown whether the effect is by impairing implantation. Although studies suggest that long periods of auditory or restraint stress may inhibit implantation in rats and mice, the exact stage of pregnancy at which stress impairs implantation is unclear. Furthermore, whether stress impairs implantation by decreasing the heparin-binding epidermal growth factor-like growth factor (HB-EGF), estrogen and/or progesterone and whether by acting on embryos or on the uterus need further investigations. In this study, a 24-h restraint stress was initiated at 15:30 of day 3 (regimen 1) or at 07:30 (regimen 2) or 15:30 of day 4 (regimen 3) of pregnancy (vaginal plug  =  day 1) to observe effects of restraint stress applied at different peri-implantation stages on implantation. Among the three regimens, whereas regimens 1 and 3 affected neither term pregnancy nor litter size, regimen 2 reduced both. Further observations indicated that regimen 2 of restraint stress also delayed blastocyst hatching and the attachment reaction, decreased serum concentrations of progesterone and estradiol, and down regulated the expression of HB-EGF in both the endometrium and blastocysts. Taken together, the results suggested that restraint stress inhibited mouse implantation in a temporal window-dependent manner and by impairing blastocyst activation and hatching and uterine receptivity via down-regulating HB-EGF, estrogen and progesterone. Thus, the stress applied within the implantation window impaired implantation by acting on both embryos and the uterus.

## Introduction

Studies suggest that psychological stress has adverse effects on reproduction in women. For example, thin women with poor psychosocial profiles are at increased risk of giving birth to low birth weight and preterm infants when depressed during pregnancy [1], and psychosocial stress during pregnancy is associated with spontaneous preterm birth and low birth weight even after adjustment for maternal demographic and behavioral characteristics [2]. In comparison to fertile controls, infertile women have been reported to have a higher incidence of personality profiles including greater suspicion, guilt and hostility and higher levels of circulating prolactin and cortisol [3], [4]. Furthermore, psychological stress among women undergoing in vitro fertilization (IVF) or gamete intra-fallopian transfer [5] has often been associated with a decrease in number of oocytes retrieved and fertilized, as well as in pregnancy rate, live birth delivery and birth weight [6].

Although IVF and embryo transfer has overcome many shortcomings of human infertility, especially oviductal and male factors, delivery rates per retrieval remain disappointingly low (10–30%), with one cause being implantation failure [7]–[9]. It is known that infertility is a source of profound psychological distress for patients [10], [11], and those who choose to undergo IVF often suffer additional anxiety and concern. For example, unsuccessful cycles and the threat of failure cause significant psychological distress [12], [13]. The costs of treatment and medical aspects of the procedures such as surgery, anesthesia and pain can also cause concern [14]–[16]. Although our previous studies have shown that restraint stress applied during oocyte pre-maturation (similar to the distress taking place during the FSH-priming period of human IVF) diminishes the developmental potential of mouse oocytes with reduced blastocyst formation and litter size [17], [18], it is not known whether the psychological stress associated with the IVF procedure would have a carrying-over effect that affects implantation directly by reducing the uterine receptivity. This question can be answered by determining the critical stage of pregnancy at which stress impairs implantation. Furthermore, although auditory stimuli at regular intervals for 48 h appeared to interfere with the implantation process in rats [19], and restraint stress on days 1–3, 4–6 or 1–6 of pregnancy reduced the pregnancy rate and average litter size of mice [20], the exact temporal window by which stress inhibits implantation and whether stress impairs implantation by acting on embryos or on the uterus remain to be specified.

Successful implantation requires reciprocal interactions between the implantation-competent blastocyst and the receptive uterus [9]. The major hormones that specify uterine receptivity are the ovarian steroids progesterone (P4) and oestrogen (E2). In addition, other mediators including cytokines, growth factors, homeotic gene products and prostaglandins participate in the implantation process in an autocrine, paracrine, or juxtacrine manner [21]. Heparin-binding epidermal growth factor-like growth factor (HB-EGF), encoded by the Hbegf gene, has been identified as an early mediator of embryo-uterine interactions during implantation [22]. Thus, HB-EGF is expressed both in the blastocyst and in the uterus during implantation of different species [23], and studies using genomic Hbegf mutant mice have shown that maternal deficiency of HB-EGF deferred on-time implantation, leading to compromised pregnancy outcome [24]. It is not known, however, whether the stress-induced implantation failure is related with a reduced expression of HB-EGF.

Restraint of animals is an experimental procedure developed for studies of psychogenic stress [25], [26]. According to Golub et al. [27], “psychogenic” refers to the implication that no invasive physical procedure or tissue trauma is involved but, rather, that the stress response is initiated in the brain by the psychological distress of being unable to move freely. Two subtypes of restraint that are often used are confinement, where movement is limited by a plastic or metal tube [28], and immobilization, where the animal's limbs and body are held immobile by tape or plaster [29]. However, metal tubes also restrict vision and light, which may reduce or enhance stressfulness [27], and tape or plaster immobilization may cause physical insults, such as impairment of blood flow. To overcome these shortcomings, we have established a new restraint system in which mice were restrained in a small, steel-wire mesh cage that was placed in their home cage with exactly the same photoperiod and controlled temperature as those of the unstressed control animals and that allowed mice to move back and forth and take food and water freely [17]. In this study, by using our restraint system that was modeling a psychological stress applied during the critical stages of implantation, we have mainly specified (i) the critical stage of pregnancy at which stress inhibits implantation (the stress-sensitive window of implantation); (ii) whether stress impairs implantation by acting on embryos or the uterus; and (iii) if stress inhibits implantation by down regulating HB-EGF, estrogen and/or progesterone.

## Results

### Effects of different regimens of peri-implantation restraint on pregnancy outcome of mice

**Figure 1:**
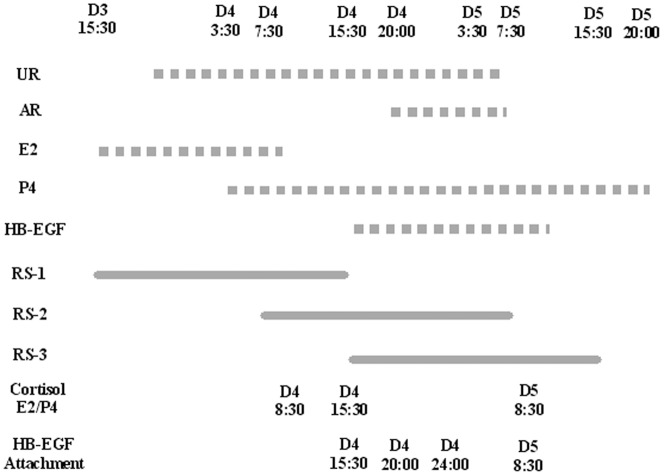
A timetable for different protocols used in the present study. Whereas reported items are shown in dotted lines, restraint regimens are shown in solid lines. Uterine receptivity (UR) is shown between 20:00 of day 3 and 07:30 of day 5, whereas attachment reaction (AR) initiates on the evening (20:00) of day 4 of pregnancy [9]. Whereas E2 peaks between 15:30 on day 3 and 08:30 on day 4, P4 peaks from 03:30 of day 4. HB-EGF begins to express about 15:30 on day 4 of pregnancy [30]. A 24-h restraint stress was started at 15:30 of day 3 of pregnancy in regimen 1 (RS-1), at 07:30 of day 4 in regimen 2 (RS-2) and at 15:30 of day 4 in regimen 3 (RS-3). Serum concentrations of cortisol, estradiol (E2) and progesterone (P4) were measured by radioimmunoassay at 1 h (08:30 of day 4), 8 h (15:30 of day 4) and 24 h (08:30 of day 5) of restraint stress with regimen 2. Observation of blastocyst hatching, attachment reaction and analysis of HB-EGF expression were performed at 8 h (15:30, day 4), 12.5 h (20:00, day 4), 16.5 h (24:00, day 4) and 25 h (08:30, day 5) after the initiation of the regimen 2 stress.

Mice that showed vaginal plugs on the same day were paired by weight and randomly assigned to control or one of the three regimens of restraint stress (Fig. 1). Rates of term pregnancy, litter sizes and birth weight of young were observed after parturition. Percentages of term pregnancy were affected by all the three restraint regimens but the effect was statistically significant only with regimen 2 (Table 1). While regimens 1 and 3 showed no effect, regimen 2 decreased the litter size significantly. None of the restraint regimens had affected the birth weight of young. Thus, pregnancy outcome of mice was significantly affected by regimen 2 of restraint stress that took place between 07:30 of day 4 and 07:30 of day 5 of pregnancy.

**Table 1:** Pregnancy outcome after mice were exposed to different regimens of restraint stress during peri-implantation.

Restraint regimens	Treatment	No. of mice showing plugs*	% Term pregnancy$	Litter size	Birth weight of pups (g)
Regimen 1	Control	24	91.7±4.2^a^	9.2±0.8^a^	1.74±0.05^a^
	Stressed	24	79.2±4.2^a^	9.0±1.1^a^	1.73±0.04^a^
Regimen 2	Control	24	95.8±4.2^a^	11.0±0.7^a^	1.80±0.03^a^
	Stressed	24	41.7±4.2^b^	4.5±1.2^b^	1.88±0.06^a^
Regimen 3	Control	24	91.7±4.2^a^	9.7±0.9^a^	1.76±0.02^a^
	Stressed	24	62.5±12.5^a^	6.7±1.2^a^	1.67±0.06^a^

a–b : Values without a common letter in their superscripts differ (P<0.05) in the same column within stress regimens.

*Each treatment was repeated 3 times and each replicate contained data from 8 mice.

$ In this paper, % Term pregnancy refers to the ratio of mice giving birth/mice showing vaginal plugs, and Independent samples t-tests were conducted using SPSS to compare the effects of different regimens of restraint stress on term pregnancy.

### Effects of peri-implantation restraint stress on attachment reaction and blastocyst hatching

Control mice and the regimen 2-stressed mice were sacrificed at 20:00 and 24:00 on day 4 and at 08:30 on day 5 of pregnancy to recover blastocysts and to observe implantation sites (IS). Both percentages of mice showing IS and the number of IS per mouse were significantly lower in stressed than in control mice at 20:00 and 24:00 on day 4, but the difference became less significant by 08:30 of day 5 (Table 2). In contrast, differences in the number of blastocysts obtained per mouse were insignificant between control and stressed mice. At 20:00 and 24:00 of day 4, the number of IS per mouse was much higher than the number of hatched blastocysts per mouse in both control and stressed mice. The percentage of hatched blastocysts was lower in stressed than control mice and the difference was significant at 24:00 of day 4. Taken together, the results suggest that the peri-implantation restraint stress delayed both embryo attachment to the uterus and blastocyst hatching and that shedding of the zona pellucida is not necessarily initiated before localized increased permeability changes occur in the uterus.

**Table 2:** Effects of regimen 2 restraint stress on attachment reaction and blastocyst hatching.

Time for examination	Treatment	Mice showing plugs*	% Mice with IS$	IS per mouse	Blastocysts per mouse	Hatched blastocyst/mouse	% Hatched/total blastocysts*
D4 20:00	Control	12	83.3±16.7^a^	6.4±1.0^a^	10.9±0.7^a^	0.7±0.3^a^	6.8±1.1(9/131)^a^
	Stressed	12	16.7±8.3^b^	1.6±1.1^b^	9.6±0.9^a^	0.4±0.2^a^	4.8±2.6(5/95)^a^
D4 24:00	Control	12	100±0.0^a^	8.9±0.7^a^	6.8±0.8^a^	3.1±0.5^a^	41.9±4.9(34/82)^a^
	Stressed	10	61.1±5.6^b^	4.5±1.3^b^	6.7±1.6^a^	1.0±0.7^b^	18.3±9.8(8/67)^b^
D5 08:30	Control	15	100±0.0^a^	11.7±0.8^a^	4.1±0.9^a^	5.5±0.7^a^	96.9±1.6(60/62)^a^
	Stressed	15	73.3±6.7^a^	8.1±1.5^a^	5.5±1.2^a^	5.0±1.3^a^	63.5±11.0(56/88)^b^

a–b : Values without a common letter in their superscripts differ (P<0.05) in the same column within time points.

*Each treatment was repeated 3 times with each replicate containing 3–5 mice.

$ % Mice with IS indicates the ratio of mice with implantation sites (IS)/mice showing vaginal plugs, and Independent samples t-tests were conducted using SPSS to locate the difference between stressed and control mice.

### Peri-implantation restraint stress decreased mouse serum concentrations of progesterone and estradiol

**Figure 2:**
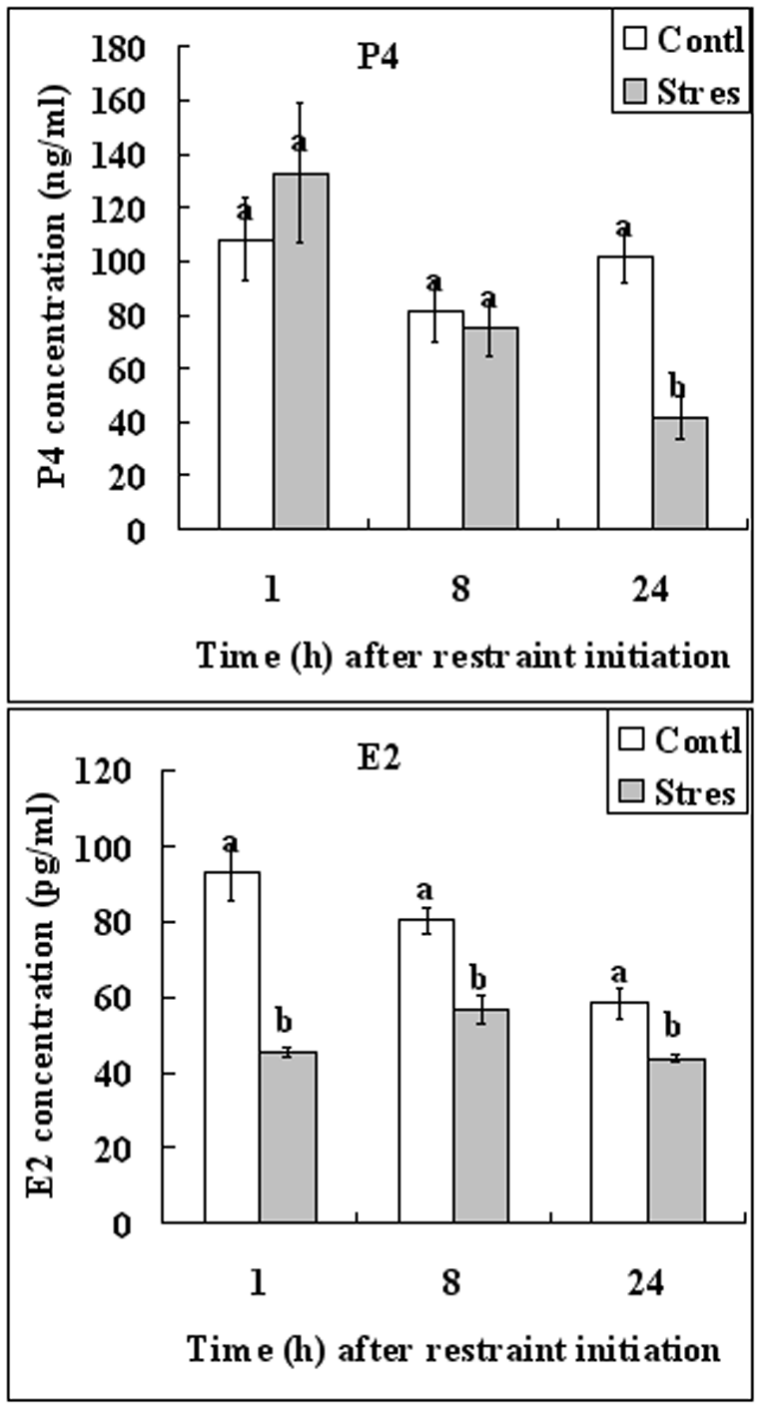
Serum concentrations of progesterone (ng/ml) and estradiol (pg/ml) after mice were exposed to regimen 2 of restraint stress for different times. a–b: Values without a common letter above their bars differ (P<0.05) between stressed (Stres) and control (Contl) mice within time points. Each treatment was repeated 5 times with each replicate containing one mouse.

At 1, 8 and 24 h after the initiation of regimens 2 restraint, blood samples were collected for radioimmunoassay of progesterone and estradiol (Fig. 1). In the unstressed control mice, whereas the P4 concentration remained constant during the 24-h period, the E2 level decreased gradually (Fig. 2). Restraint stress decreased the level of P4 significantly at 24 h after restraint initiation while it reduced the E2 level at all the three time points examined.

### Peri-implantation restraint stress down regulated the expression of HB-EGF in both the uteri and blastocysts

**Figure 3:**
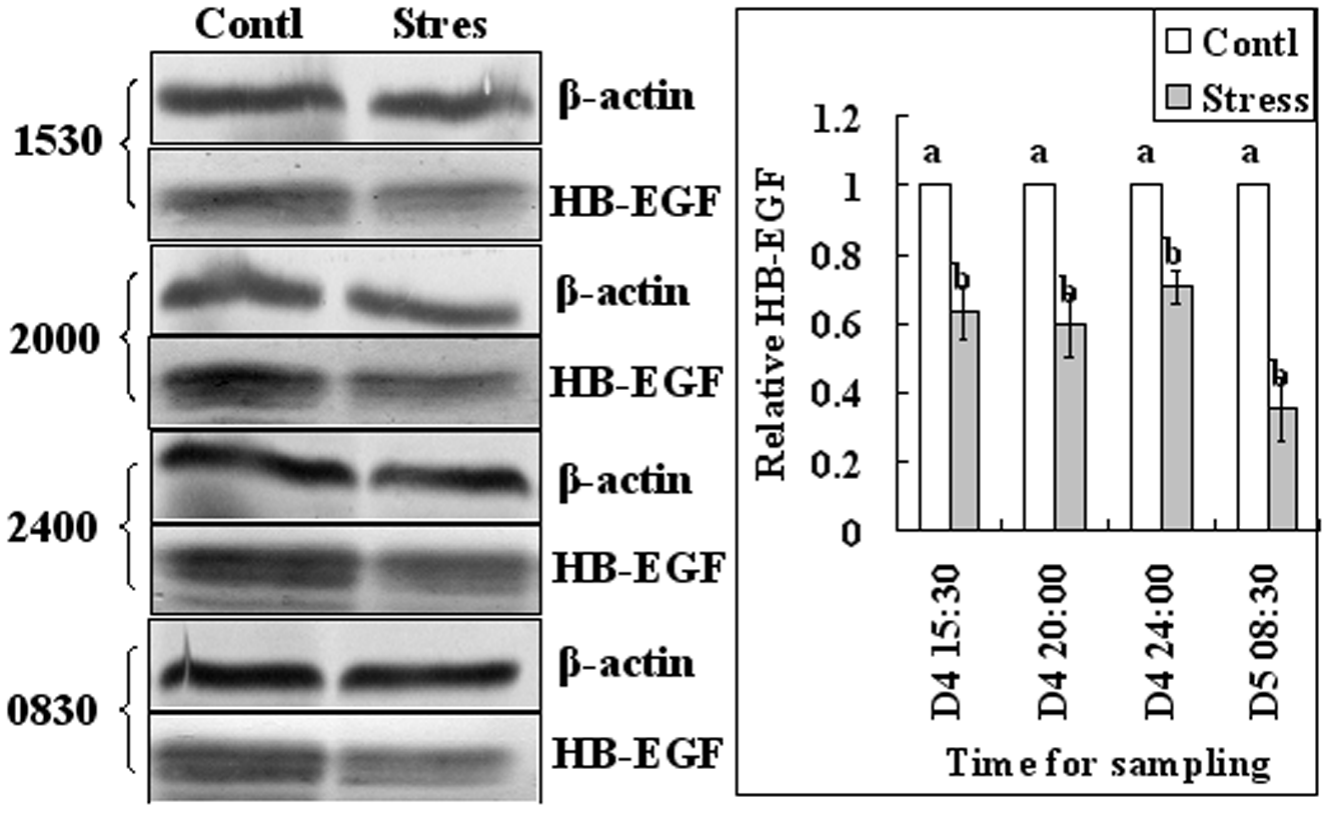
Relative levels of HB-EGF in endometrium of control (Contl) mice and mice stressed with regimen 2 (Stres). Results of Western blot analysis. Endometrium samples were collected for western analysis at 15:30, 20:00 and 24:00 of day 4 and at 08:30 of day 5. On each experimental day, the value of control mice was set as one and the value of stressed mice was expressed relative to this value. a,b: Means with a different letter above their bars differ significantly (P<0.05).

**Figure 4:**
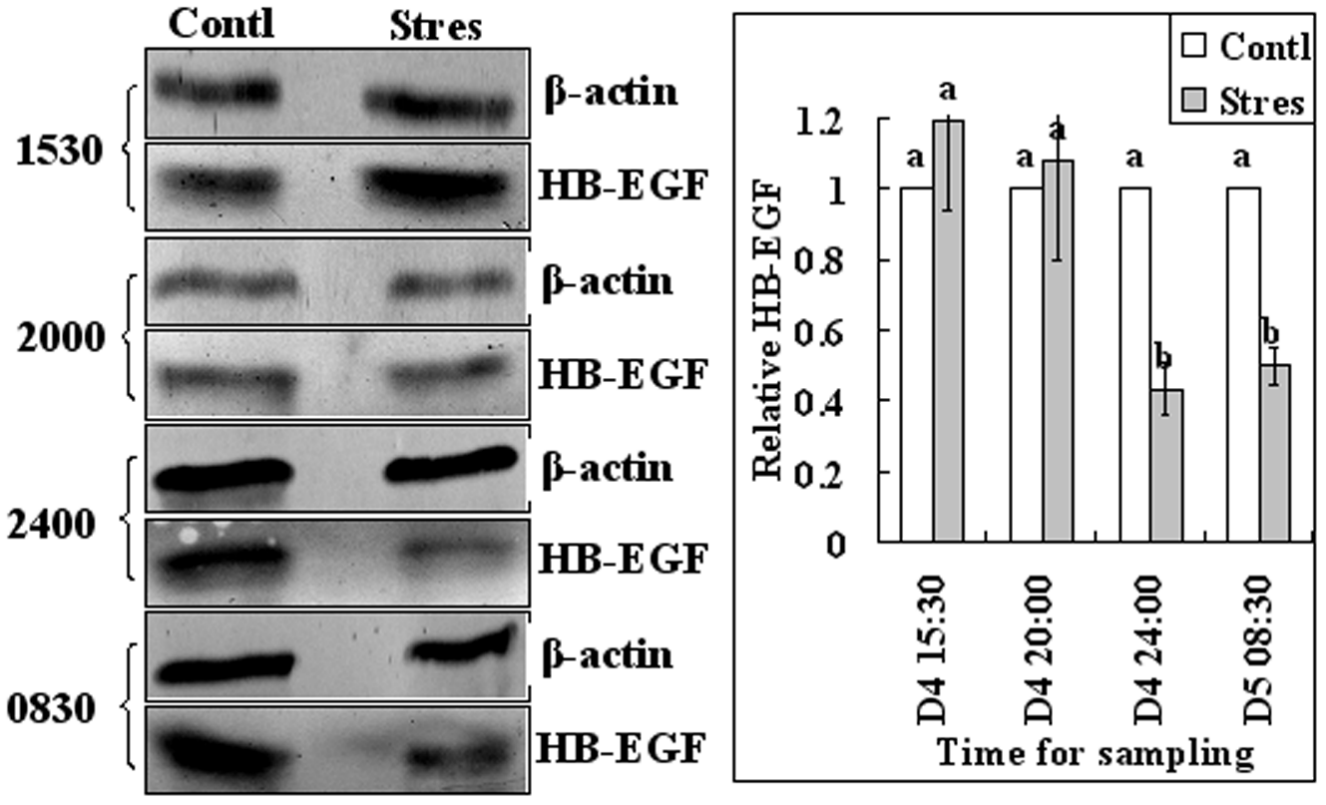
Relative levels of HB-EGF in blastocysts of control (Contl) mice and mice stressed with regimen 2 (Stres). Results of Western blot analysis. Blastocysts were collected for western analysis at 15:30, 20:00 and 24:00 of day 4 and at 08:30 of day 5. On each experimental day, the value of control mice was set as one and the value of stressed mice was expressed relative to this value. a,b: Means with a different letter above their bars differ significantly (P<0.05).

Since it was reported that HB-EGF first appears in uterine epithelial cells juxtaposed with blastocysts around 16:00 on day 4 of pregnancy [30], endometrium and blastocysts samples were collected from control and regimen 2-stressd mice for western analysis of HB-EGF at 15:30, 20:00 and 24:00 of day 4 and at 08:30 of day 5 (Fig. 1). Results showed that the relative levels of HB-EGF in endometrium were significantly lower in stressed mice than in control mice at all time points examined (Fig. 3). The level in blastocysts did not differ between stressed and control mice at 15:30 and 20:00 of day 4 but decreased significantly in stressed mice by 24:00 of day 4 and 08:30 of day 5 (Fig. 4). Results suggested that the HB-EGF expression was affected by stress earlier in the uterus than in embryos.

### Effects of peri-implantation restraint stress on serum cortisol concentrations of mice

**Figure 5:**
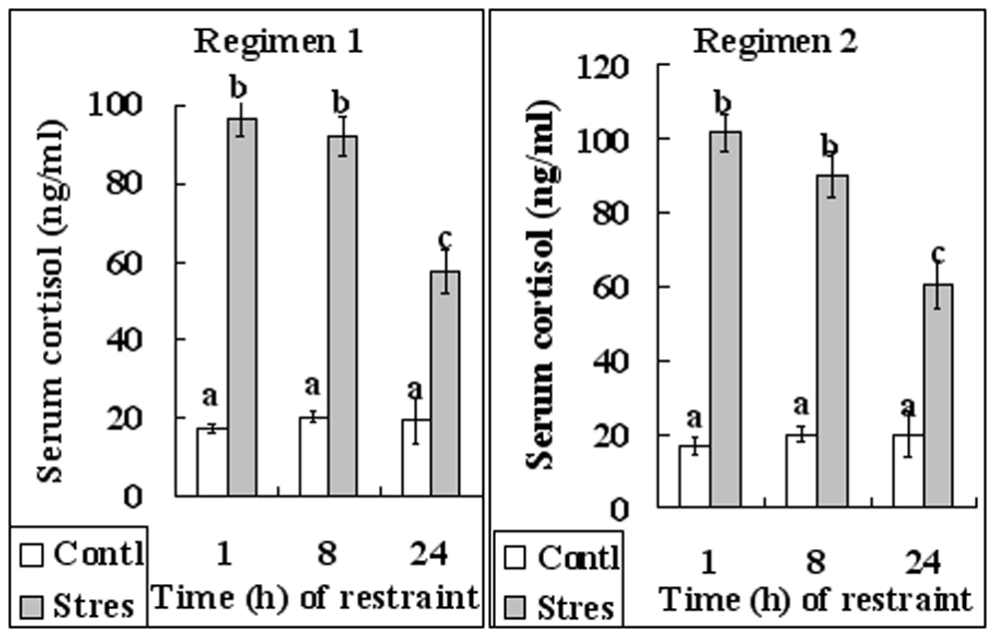
Serum cortisol concentrations (ng/ml) in unstressed control (Contl) mice and mice that were exposed to restraint stress of regimens 1 or 2 for different times. a–c: Values with different letters above their bars differ (P<0.05).

To evaluate the stress response of mice to our restraint system, serum concentrations of cortisol were measured by radioimmunoassay. Blood samples were collected at 1, 8 and 24 h after the initiation of restraint by regimens 1 and 2 (Fig. 1). Whereas the cortisol level in control mice did not change significantly across the 24 h period, cortisol concentrations in mice stressed by either regimen 1 or regimen 2 increased significantly and peaked at 1 h and 8 h after the initiation of restraint (Fig. 5). Cortisol concentration in stressed mice declined thereafter but remained significantly higher than that of their unstressed counterparts at 24 h of restraint. The results suggested that our restraint regimens consistently stressed the animals.

### Effects of peri-implantation restraint stress on food and water intake of mice

To test whether our restraint system would affect feeding and if a food and water deprivation control would be necessary for experiments, the restrained and control mice were individually kept for 24 h in cages with the floor covered by a pressboard. Food (including that crushed on the floor) and water were weighed both before and after experiments. The results indicated that the average intake of food and water did not differ significantly between unrestrained control mice and the mice restrained with either regimen 1 or regimen 2 (Table 3).

**Table 3:** Food and water intake of mice during restraint stress of different regimens in the peri-implantation period.

Stress regimens	Treatment	No. of mice*	Food (g)	Water (ml)
Regimen 1	Control	14	6.12±0.24^a^	6.70±0.11^a^
	Stressed	14	6.24±0.17^a^	6.75±0.18^a^
Regimen 2	Control	12	5.79±0.30^a^	6.80±0.40^a^
	Stressed	12	5.95±0.22^a^	6.76±0.16^a^

a–b : Values without a common letter in their superscripts differ (P<0.05) in the same column within stress regimens.

*Each treatment was repeated 3 times with each replicate containing 4–5 mice.

## Discussion

Among the three restraint regimens tested in this study, whereas regimen 1 and regimen 3 affected neither term pregnancy nor litter size, regimen 2 reduced both. Regimen 2 of restraint stress also delayed embryo attachment to the uterus and blastocyst hatching. Why was regimen 2 the most disruptive to mouse embryo implantation? First, regimen 2 was the one that covered the most part of the implantation window and started long before the initiation of the attachment reaction, among the three regimens (Fig. 1). Previous studies indicated that the window of implantation is very narrow and is under strict regulation by ovarian hormones [31]. It is believed that in rodents the window of receptivity lasts for about 24 h, after which the uterus proceeds to non-receptivity [32]. In the mouse, the first discernible sign of implantation is an increased uterine stromal vascular permeability at the site of blastocyst apposition [33], which coincides with the attachment reaction between the blastocyst and the uterine luminal epithelium. The attachment reaction occurs in the mouse around midnight on day 4 of pregnancy [34], [35]. According to a review by Wang and Dey [9], mouse uterine receptivity occurs between 20:00 of day 3 and 07:30 of day 5, whereas attachment reaction initiates at 20:00 on day 4 of pregnancy.

Second, the present results indicated that regimen 2 restraint significantly decreased serum concentrations of progesterone and estradiol, and down regulated the expression of HB-EGF in both the endometrium and blastocysts. Restraint stress during pregnancy was found to be luteolytic, decreased serum P4 concentrations and caused fetal loss in rats [36] and mice [20], although similar effects on E2 have not been reported. During normal pregnancy, E2 peaks around 15:30 on day 3 until 08:30 on day 4 of pregnancy, and P4 peaks from 03:30 of day 4 onwards [9]. Das et al. [30] demonstrated that the HB-EGF gene was expressed in the mouse uterine luminal epithelium surrounding the blastocyst 6–7 h before the attachment reaction that occurs at 22:00–23:00 h on day 4 of pregnancy. Similarly, Zhang et al. [43] reported that the HB-EGF expression in the uterine epithelial cells was essential for the upcoming stromal decidualization. This suggested that the HB-EGF expression revealed by our Western analysis on the endometrium came mainly from the epithelial cells. Thus, whereas regimen 2 inhibited all the rises of E2, P4 and HB-EGF, regimen 1 would have missed HB-EGF while regimen 3 would have missed E2 (Fig. 1). In all mammals studied, P4 is essential for the maintenance of early pregnancy and in most species, it is also a pre-requisite for implantation [37]. In the rat and mouse, post-ovulatory estrogen is also required to achieve an endometrial status conducive both to implantation and to normal blastocyst development [38]. In addition, HB-EGF has been identified as an early mediator of embryo-uterine interactions during implantation [22], and studies using genomic Hbegf mutant mice have shown that maternal deficiency of HB-EGF deferred on-time implantation, leading to compromised pregnancy outcome [24].

The present results demonstrated that the regimen-2 restraint stress postponed both blastocyst hatching and the attachment reaction between the blastocyst and the uterus. Meanwhile, it decreased the levels of E2 and P4 in serum and that of HB-EGF in both the endometrium and the blastocyst. It is known that only activated competent blastocysts can induce attachment reaction and that only the hatched blastocysts can implant into the uterus. In other words, the present results suggested that restraint stress postponed attachment reaction by inhibiting blastocyst activation and it impaired implantation by preventing blastocyst hatching. Maternal steroids have been shown to regulate genital tract secretions in vivo [38] and could by this means exert an influence on the developing embryo. As reviewed by Sauer [39], the shedding of the zona pellucida involves the action of proteolytic enzymes arising from the endometrium, which seem to be controlled by the ovarian steroids. It has been reported that E2 induces very rapidly, but transiently, a factor(s) in the P4-primed uterus that activates the dormant blastocysts in utero for implantation in the receptive uterus [32]. Furthermore, there is also evidence that catecholestrogens that are produced from primary estrogens in the uterus activate blastocysts [40].

Using a delayed-implantation mouse model, a global gene-expression study showed that activation or dormancy states of the blastocyst are molecularly distinguishable [23]. The same study also showed an up-regulated expression of HB-EGF gene in activated blastocysts, a finding that is complementary to earlier reports of up-regulated expression of its receptors ErbB1 and ErbB4 in similar blastocysts [41], [42]. Furthermore, in vitro studies showed that HB-EGF induced auto-phosphorylation of blastocyst EGF receptor, and promoted blastocyst growth, zona-hatching and trophoblast outgrowth [30]. Taken together, the present results suggested that the peri-implantation restraint stress inhibited blastocyst activation and hatching by reducing the levels of estrogen, progesterone and HB-EGF, leading to implantation failure due to missing of the right implantation window. However, although the above data suggest a leading role for the embryo in implantation, the present results that the expression of HB-EGF in the endometrium decreased earlier than that in blastocysts following restraint stress emphasize that restraint stress inhibited implantation (particularly the early attachment reaction) by affecting the uterus as well as the blastocysts.

In summary, although it is known that psychological stress affects reproduction in women, whether the effect is by impairing implantation is unclear. Although studies suggest that long periods of auditory or restraint stress may inhibit implantation, the exact stage of pregnancy at which stress impairs implantation remains to be determined. Furthermore, whether stress impairs implantation by decreasing HB-EGF, estrogen and/or progesterone and whether it does so by acting on embryos or on the uterus need further investigations. The present study has investigated these questions by using a mouse restraint system that was modeling a psychological stress applied during the critical stages of implantation. The results indicated that restraint stress inhibited mouse implantation in a temporal window-dependent manner (with the implantation window being the most susceptive to stress) and by impairing blastocyst activation and hatching and uterine receptivity via down-regulating HB-EGF, estrogen and progesterone. Thus, stress applied during the implantation window impaired implantation by acting on both embryos and the uterus.

## Materials and Methods

### Ethics Statement

Mouse care and use were conducted exactly in accordance with the guidelines and approved by the Animal Research Committee of the Shandong Agricultural University, P. R. China (Permit number: 20010510). According to the guidelines of the committee, the animal handling staff (including each post-doc, doctoral or masters student) must be trained before using animals. Mice must be housed in a temperature-controlled room with proper darkness-light cycles, fed with a regular diet, and maintained under the care of the Experimental Animal Center, Shandong Agricultural University College of Animal Science and Vet Medicine. In the present study, mice were sacrificed by cervical dislocation. The only procedure performed on the dead animals was the collection of oocytes from the ovaries.

Unless otherwise specified, all chemicals and reagents used in the present study were purchased from Sigma Chemical Co. (St. Louis, MO, USA).

### Mice and restraint treatment

Mice of the Kunming breed were kept in a room with a constant temperature (22–25°C) and 14 h/10 h light-dark cycles, the dark starting at 8 pm. Female and male mice were used at the age of 8–10 weeks and 10–12 weeks, respectively. Virgin females were placed with males and checked daily at 07:30 for copulatory plugs. Upon detection of a vaginal plug (day 1 of pregnancy), females were randomly assigned to restraint treatments or as controls.

For restraint treatment, an individual mouse was put in a micro-cage constructed by the authors [17], which was placed in an ordinary home cage. The micro-cage offered the same photoperiod and controlled temperature as in the large home cage for the unstressed animals. While in the micro-cage, mice could move back and forth to some extent and could take food and water freely, but they could not turn around. Control mice remained in their cages with food and water during the time treated females were stressed. Three regimens of 24-h restraint stress were adopted: regimen 1 took place from 15:30 of day 3 to 15:30 on day 4 of pregnancy, regimen 2 was applied between 07:30 on day 4 and 07:30 of day 5, and regimen 3 was between 15:30 of day 4 and 15:30 of day 5 (Fig. 1).

### Determination of pregnancy outcome

Mice that showed vaginal plugs on the same day were paired by weight and randomly assigned to a control or one of the three regimens of restraint stress (Fig. 1). Rates of term pregnancy, litter sizes and birth weights of young were determined after parturition.

### Recovery and counting of blastocysts

At different times of pregnancy, control and stressed mice were sacrificed and their uteri were flushed with M2 medium for blastocysts. The blastocysts obtained were classified under a stereo microscope as hatched or not hatched according to the existence of an intact zona pellucida.

### Determination of implantation sites

To determine the implantation sites (IS), control and stressed mice were injected intravenously (via tail vein) with 0.1 ml of 1% Chicago blue B in saline at different times of pregnancy. The injected animals were sacrificed 5 min later and blue bands around the uterine horns that indicated initiation of the implantation process were recorded.

### Hormone assay

Mice were killed by decollation. Trunk blood (about 1 ml) was collected into ice-cooled centrifugal tubes and centrifuged (1700×g, 10 min, 4°C) to separate serum. The serum collected was stored at −80°C until hormone assay. Radioimmunoassay was performed by the Central Hospital of Tai-An City using commercial kits from Jiuding Biomedical Techniques Co. Ltd., Tianjin, China. The minimum levels of detection for assays of estradiol (E2), progesterone (P4) and cortisol were 1 pg/ml, 1 ng/ml and 10 ng/ml, respectively. The intra- and inter-assay coefficients of variation were 7.7% and 8.9% for estradiol, 7.2% and 8.9% for progesterone, and <10% and <15% for cortisol.

### Western blot analysis

Endometrium was separated from myometrium and was snap-frozen in liquid nitrogen. Endometrium from three animals for each treatment group was pooled and three sets were used for each treatment. The frozen tissue were transferred to a glass homogenizer with extraction buffer (50 mM Tris-HCI, pH 7.5; 150 mM NaCl; l% Triton X-100; 0.25% sodium deoxycholate and 1 mM Phenylmethylsulphonyl fluoride) and homogenization was done while cooling on ice. The homogenates were then centrifuged (20000×g, 10 min, 4°C), and the supernatant was collected. After the total protein concentration was determined by using the bicinchoninic acid (BCA) kit (P0010S, Beyotime Institute of Biotechnology, Haimen City, China) and adjusted to 1 µg/µl, 20 µl sample containing 20 µg protein of each microfuge tube was frozen at −80°C until use. Blastocysts (n = 60) were placed in a 1.5-ml microfuge tube containing 20-µl sample buffer (20-mM Hepes, 100-mM KCl, 5-mM MgCl_2_, 2-mM DTT, 0.3-mM phenylmethyl sulfonyl fluoride, 3 µg/ml leupetin, pH 7.5) and frozen at −80°C until use.

For protein extraction, 6.66 µl of 4×SDS-PAGE loading buffer (P1015, Solarbio) was added to each tube, and the tubes were heated to 100°C for 5 min. Total proteins were separated on a 15% polyacrylamide gel by SDS-PAGE and transferred electrophoretically on to PVDF membranes. The membranes were washed twice in TBST (150 mM NaCl, 2 mM KCl, 25 mM Tris, 0.05% Tween 20, pH 7.4) and blocked for 1 h with TBST containing 3% BSA at room temperature. The membrane was then incubated overnight at 4°C with mouse anti-HB-EGF antibody (1∶200 dilution, sc-74441, Santa Cruz Biotechnology) and mouse anti-β-actin antibody (1∶6000 dilution, AICM001, Beijing 4A Biotech Co., Ltd.). After being washed 3 times in TBST (5 min each), the membranes were incubated for 1 h at 37°C with alkaline phosphatase-conjugated horse anti-mouse IgG (1∶600 dilution, ZB-2310, ZSGB-Biotechnology, Beijing, China). Finally, signals were detected by a BCIP/NBT alkaline phosphatase color development kit (C3206, Beyotime Institute of Biotechnology, Haimen City, China). Relative quantities of protein were determined with Image-Pro plus software by analyzing the sum density of each protein band image. The relative quantity values of HB-EGF in unstressed control mice were arbitrarily set as one and the values in stressed mice were expressed relative to this quantity.

### Data analysis

There were at least three replicates for each treatment. In all experiments but the ones for percentage term pregnancy and percentage mice with implantation sites, data were arc sine transformed and analyzed with ANOVA; a Duncan multiple comparison test was used to locate differences. In the experiments for percentage term pregnancy and percentage mice with implantation sites, independent samples t-tests were conducted to compare the effects between unstressed control and the stressed mice. The soft ware used was SPSS (Statistics Package for Social Science). Data are expressed as mean ± SE, and P<0.05 was considered significant.
